# An Uneven Node Self-Deployment Optimization Algorithm for Maximized Coverage and Energy Balance in Underwater Wireless Sensor Networks

**DOI:** 10.3390/s21041368

**Published:** 2021-02-15

**Authors:** Luoheng Yan, Yuyao He, Zhongmin Huangfu

**Affiliations:** 1 School of Marine Science and Technology, Northwestern Polytechnical University, Xi’an 710072, China; heyyao@nwpu.edu.cn; 2 College of Information Engineering, North China University of Water Resources and Electric Power, Zhengzhou 450046, China; huangpu@ncwu.edu.cn

**Keywords:** underwater wireless sensor network, depth-adjustment, self-deployment, coverage rate, growth ring, energy balance

## Abstract

The underwater wireless sensor networks (UWSNs) have been applied in lots of fields such as environment monitoring, military surveillance, data collection, etc. Deployment of sensor nodes in 3D UWSNs is a crucial issue, however, it is a challenging problem due to the complex underwater environment. This paper proposes a growth ring style uneven node depth-adjustment self-deployment optimization algorithm (GRSUNDSOA) to improve the coverage and reliability of UWSNs, meanwhile, and to solve the problem of energy holes. In detail, a growth ring style-based scheme is proposed for constructing the connective tree structure of sensor nodes and a global optimal depth-adjustment algorithm with the goal of comprehensive optimization of both maximizing coverage utilization and energy balance is proposed. Initially, the nodes are scattered to the water surface to form a connected network on this 2D plane. Then, starting from sink node, a growth ring style increment strategy is presented to organize the common nodes as tree structures and each root of subtree is determined. Meanwhile, with the goal of global maximizing coverage utilization and energy balance, all nodes depths are computed iteratively. Finally, all the nodes dive to the computed position once and a 3D underwater connected network with non-uniform distribution and balanced energy is constructed. A series of simulation experiments are performed. The simulation results show that the coverage and reliability of UWSN are improved greatly under the condition of full connectivity and energy balance, and the issue of energy hole can be avoided effectively. Therefore, GRSUNDSOA can prolong the lifetime of UWSN significantly.

## 1. Introduction

Acoustic communication is the main mode for UWSNs [1,2,3]. In recent years, acoustic-based UWSNs have been applied in lots of fields such as environment monitoring, military surveillance, data collection, etc [4,5,6]. Research on UWSNs mainly involves node deployment [7,8,9,10], node localization [11], network protocol design [3,12], simulation [13,14,15], etc. Deployment of sensor nodes in 3D UWSNs is a crucial issue, which directly affects the performance of networks. However, there is still a large gap between the current research and practical application for this problem [16]. According to the mobility characteristics of sensor nodes, the current deployment schemes can be classified into three categories in general: static deployment, self-deployment and movement-assisted deployment [8]. Static deployment is one of the main deployment approaches, in which sensors are fixed one-by-one manually to their precomputed positions [17]. Although this deployment method reduces the complexity in some special scenarios, its process is very time consuming and costly, especially for a large volume monitored area. Moreover, static deployment is infeasible for some inaccessible regions during military surveillance and pollution detection applications. Movement-assisted deployment is another main mode of deployment for UWSNs. The sensors are embedded in mobile platforms such as autonomous underwater vehicles (AUVs). Driven by these devices, the sensors can move to their final locations after they are dropped randomly onto the water surface [18,19,20]. Assisted by these mobile devices, sensors can move freely to any location in the underwater region [21,22] to improve network coverage and connectivity. However, this deployment method is not practical because these types of sensors are always large and costly. Therefore, finding the low-cost and high-coverage rapid deployment approach for UWSNs has attracted attention from lots of scholars and engineers in recent years.

In order to tackle the problem mentioned above, one of the feasible solutions is the self-deployment method, that is, autonomous depth-adjustment deployment for sensors which have the restricted vertical moving capability [23]. For this scheme, the sensor nodes are dropped randomly onto the water surface initially, so that the coverage is highly uncertain. Then, the sensors dive to their final positions adjusting their depths by themselves. Compared with the AUV-assisted node deployment mentioned above, this depth-adjustment node self-deployment is practical because it relatively simple, readily available, and economic in engineering application. However, due to the complex underwater environment, it is challenging to achieve the optimal depth-adjustment deployment with the goal of maximized coverage, minimized energy consumption and guaranteed connectivity to sink nodes.

Thus, a growth ring style uneven node depth-adjustment self-deployment optimization algorithm (GRSUNDSOA) for UWSNs is proposed in this paper. Initially, the sensors are scattered to the water surface to form a connected network on this 2D plane. Then, starting from the sink node, a growth ring (GR) style increment strategy is utilized to organize the common nodes as tree structures and the depths for all nodes are computed based on the global maximizing coverage utilization and energy balance iteratively. Finally, all the nodes dive to the new positions once according to the computed depths and a 3D connected UWSN with uneven distribution nodes and balanced energy is constructed. Our contributions in this paper are described as follows:

(1) A growth ring style-based scheme is proposed for constructing a connective tree structure. In this scheme, an incremental broadcast radius calculation is utilized to determine the GRs and construct the connective tree structure. It means that the radial distance between the GRs increases with its distance to sink node increasing. The nodes of UWSNs constructed by this deployment method are non-uniformly distributed ring by ring. That is, the network deployed by this strategy has the following characteristics: The number of nodes near sink node is large and the nodes distribution is dense, on the contrary, the number of nodes far from sink node is small and the nodes distribution is sparse. This conforms to the hot spot characteristics near the sink node. Thus, it has better network reliability, and its energy consumption is more balanced. It can avoid the energy hole [24,25] effectively.
(2) A novel depth-adjustment self-deployment algorithm based on growth ring style is proposed. This algorithm strives to obtain the optimal network coverage rate on the basis of ensuring energy consumption balance and connectivity. Different from the recent approaches in the literature such as CDA [26,27], Cacc [28], VODA [29], and DODA [30], the proposed algorithm searches the global optimal dive position based on the basic nodes for depth calculation rather than the local optimal dive position. Furthermore, the proposed depth calculation method is with the goal of comprehensive optimization of both maximizing coverage utilization and energy balance, rather than just coverage only. Therefore, the coverage rate and energy balance of UWSN deployed by GRSUNDSOA is improved greatly.

The rest of the paper is organized as follows: Related works are addressed in Section 2. Section 3 describes our problem definition, model and proposal. The proposed algorithm is presented in Section 4 in detail. Simulation and analysis are provided in Section 5. Finally, conclusions are given in Section 6.

## 2. Related Research

As mentioned above, in recent years, research on UWSNs mainly involves topics like node deployment [7,8,9,10], node localization [11], network protocol design [3,12], simulation [13,14,15], etc. Specially, the issue of node deployment has attracted attention from lots of scholars and engineers.

For static node deployment scheme, Alam [31] studied a series of node placement strategies with the minimum number of nodes to obtain 100% sensing coverage. In this scheme, a matric called volumetric quotient was defined. The higher the volumetric quotient is, the smaller the number of nodes requires for full 3D coverage. The simulation results show that the truncated octahedral cell has the best coverage. This method is a deterministic rule deployment, which requires precise location of sensors. However, node localization [11] is a tedious process calculated by GPS, which is very time consuming and costly.

Senouci et al. [18] presented a survey and classification about the movement-assisted sensor deployment algorithms. Six classes of approaches are identified based on relocation schemes. Each of these approaches adopts a specific principle to relocate the nodes from their initial position to a new target position. These mobile sensors driven by mobile device, such as AUV, are always costly. Therefore, it is unrealistic to use many mobile nodes for network deployment. In practice, the mobile sensors are always applied to patch the coverage holes. Wang et al. [19] proposed a mobile assisted hole patching scheme for hybrid WSNs based on particle swarm optimization (PSO). Some mobile nodes are utilized to repair coverage holes, and PSO algorithm is used to calculate moving position of mobile nodes. Kadu [20] proposed a movement-assisted coverage improvement approach for hole healing called Modified Hole Detection and Healing (MHDH) to recover the coverage holes.

Recently, move-restricted node self-deployment has been studied by many scholars. Detweiler et al. [23] developed a depth adjustment platform called AQUANODE. The system nodes are deployed on the water surface and connected to the water AQUANODE sensor node. A series of experiments are performed in 50 m water depth to validate this system effectiveness. Two special applications are also discussed to introduce the utilization of this system. In its experiments, the communication between sensor nodes must be through the buoys on the water surface, not the underwater acoustic communication method.

Senel et al. [26,27] proposed a self-deployment scheme named as Depth Computation Approach (CDA). In this approach, the connected paths of network are constructed using a node set called connected dominating set (CDS) to adjust the node depths. The CDS is determined based on a connected backbone constructing. The depths of dominator and non-dominator nodes are then optimized iteratively. This algorithm has relatively good performance on coverage and connectivity. However, this algorithm has poor energy consumption balance performance especially in the situation of sparse node distribution. Moreover, in the process of determining the depth of nodes, CDA can only find the local optimal solution instead of the global optimal solution due to its limitation when considering coverage redundancy; hence the coverage rate of UWSNs can be improved furtherly.

Senel [28] also proposed a self-deployment scheme named coverage-aware connectivity-constrained (Cacc) unattended sensor deployment. In this algorithm, the ground control station initially forms a 2D connected tree structure for all nodes. Then, the ground control station calculates the depth of each node one by one to reduce the coverage overlap between adjacent nodes while maintaining the network connectivity. Similarly, this depth-adjustment algorithm always calculates a local optimum for the diving depth, not the global optimum.

Su et al. [29] proposed a Voronoi-based Optimized Depth Adjustment (VODA) deployment scheme to deploy sensor nodes in a target water space. This algorithm has the goal of coverage and connectivity joint optimization. Some leader nodes are chosen to remain on the water surface based on a Voronoi diagram; on the contrary, the other nodes calculate their depths aiming to optimize coverage overlap between the nodes. This algorithm can achieve good coverage and connectivity performance. However, it needs to collect accurate position coordinates of sensor nodes when establishing the Voronoi diagram. The node localization process is always tedious and costly as mentioned above. Moreover, it does not consider the issue of energy balance and energy holes. Jin et al. [30] proposed a deployment optimization algorithm using depth adjustable nodes in underwater acoustic networks (DODA). This algorithm considers node drift situations when optimizing the node position. With the goal of maximizing network coverage, and combining the location information of neighboring nodes, this paper uses a greedy algorithm to solve the problem of node deployment optimization under the premise of keeping network connectivity. Similarly, this algorithm does not consider the issue of energy balance.

Jiang [32] presented a node self-deployment algorithm based on an uneven cluster with radius adjusting. Firstly, the nodes randomly scattered on the water surface are non-uniformly clustered according to the distance from the sink node. Each cluster head node then constructs a connected path to sink node. Finally, the depths of each node are adjusted with the goal of minimizing the coverage redundancy. This method takes the issues such as network reliability and energy consumption balance into account, however, its depth adjustment strategy cannot reach the global optimum.

Wang [33] presented a depth-adjustment scheme based on Voronoi diagrams. All move-restricted sensor nodes are firstly placed randomly on water surface. Then the special nodes with adjustable depth are found out based on Voronoi diagrams and moved vertically to the determined depth. Therefore, the deployment process is simplified due to the divided different layers with this scheme. However, the construction process of Voronoi diagrams is complicated and costly. Pompili et al. [34] proposed different deployment strategies for 2D and 3D communication architectures for UWSN. Jiang [35] proposed a depth adjustment deployment algorithm based on a 2D convex hull and spanning tree for achieving full network connectivity. Akkaya et al. [36] presented a distributed self-deployment approach in UWSNs to find its maximum coverage rate. On the contrary, in [33] the move-restricted nodes are placed on the water bottom firstly. The graph coloring-based strategy is utilized when the nodes depths are determined. This algorithm can achieve relatively good coverage and connectivity performance with appropriate parameter values. Nevertheless, its energy consumption for node deployment is too large. 

Du [37] presented the approach based on virtual force for depth-adjustment of sensor nodes. This algorithm simplifies virtual force as the repulsion force only between neighbor nodes and utilizes the virtual force to adjust the node to the position where the resultant force is zero. It only considers the coverage rate of network but ignores the connectivity rate for network performance.

## 3. Preliminaries for GRSUNDSOA

As mentioned in Section 2, most of the recent depth-adjust self-deployment algorithms only regard coverage or connectivity as the main performance indicators for network deployment. The network formed by these methods is a generally uniformly distributed 3D topology of nodes. In UWSN, the data collected by sensor nodes are transmitted to sinks via one or multi-hops from bottom to top. Hence, the nodes near the sink have a heavier data transmission load and more energy consumption than that of the other nodes. Thus, the energy non-replenishment of underwater nodes [38,39] leads to faster energy exhaustion and faster failure for nodes near sink than that of the other nodes. As a result, the data uploaded from remote nodes which have longer lifetime cannot be transferred to sinks. This will lead to an early end of the whole network life although lots of remote nodes are still in working mode. Therefore, in order to improve the quality of service (QoS) of UWSN, solving this energy hole problem caused by the imbalance of node energy consumption is also an important indicator for nodes deployment. 

Based on the above analysis, a scheme of uneven distribution node deployment, which has higher density nodes near sink and lower density nodes far from sink, is helpful to avoid the issue of energy consumption imbalance. For this scheme, there are more nodes near sink to complete data transmission and share the communication energy consumption, so that the communication energy consumption for each node in this area will be relatively reduced. Thus, the communication energy consumption of each node in the entire UWSN tends to be balanced. According to the above analysis, in this paper, a growth ring style uneven node depth-adjustment self-deployment optimization algorithm for UWSNs is proposed.

### 3.1. Network Scenario

**Figure 1:**
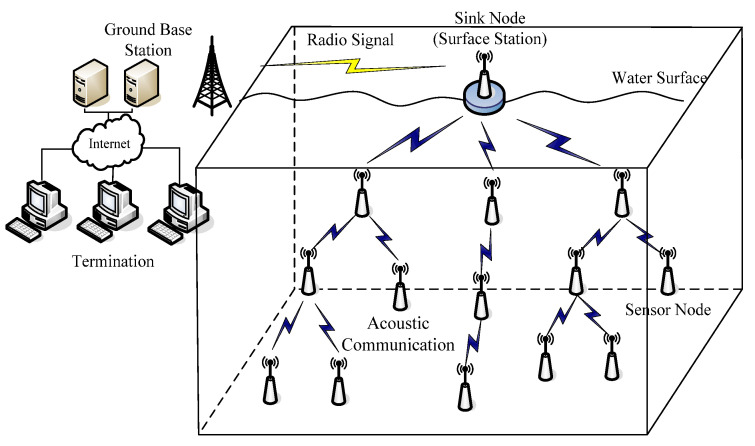
Considered 3D network scenario.

It is assumed that the nodes are scattered to the water surface and initially form a connected network on this 2D plane. These nodes have perception capabilities and restricted mobility, i.e., moving only in vertical direction by adjusting their depth via a mechanism described in [28]. This depth adjustment system is a mechanism which is composed of a motor, gearhead, timing belt and a magnetic coupler [28]. The timing belt is attached to an underwater sensor node and the magnetic coupler turns a spool of anchor line. The considered network scenario in this paper is shown in Figure 1.

Each sensor node on the water surface is moved vertically to form this 3D network and probes its vicinity in the sensing range. These nodes send their collecting data to the sink node, which is fixed on the surface station, via a one or multi-hop path through acoustic communication channels. On the contrary, the sink node maintains connectivity with the ground base station through radio.

Some assumptions are considered:

(1) In the initial phase, sensors are dropped randomly to water surface of the monitoring area, the volume of which is denoted as *Length* × *Width* × *Height* (m^3^), so that a densely populated 2D connected network is formed. The number of sensors is denoted as *N*. All nodes are in sleep mode. For each node, its coordinate position on water surface can be calculated using location algorithm.
(2) The energy of sink node is considered that can be replenished. And in this system, it is always located in the center of the monitoring area. There is only one sink node in this scenario.
(3) Each node has a unique ID. Its communication radius is denoted as *Rb* which can be adjusted but must meet the condition *Rb* ≤ *Rc*_max_, where *Rc*_max_ is denoted as the maximum communication radius. The sensing radius of each sensor is denoted as *Rs*.

### 3.2. Coverage Model

Let each sensor node in this system be denoted as *Ns_i_*, *I =* 1, 2, …, *N*, and its position coordinate in monitoring area is **X**(*Ns_i_*) = (*x_i_,y_i_,z_i_* ). Let *U*(*x,y,z*) be a pixel in monitoring area. The probability that *U*(*x,y,z*) is covered by node *Ns_i_* is denoted as *P*_cov_(*x,y,z,Ns_i_* ), which can be calculated with Equation (1) according to the Boolean perception model:

(1) $$p_{\mathrm{cov}}(x,y,z,Ns_{i})=\left\{ \begin{array}{l} 0,dist(U,Ns_{i})>R_{s}\\ 1,dist(U,Ns_{i})\leq R_{s}\; \end{array}\right.$$

where, *U* represents a pixel in monitoring area, *dist*(*U,Ns_i_*) represents the Euclidean distance between *U* and *Ns_i_*.

**Figure 2:**
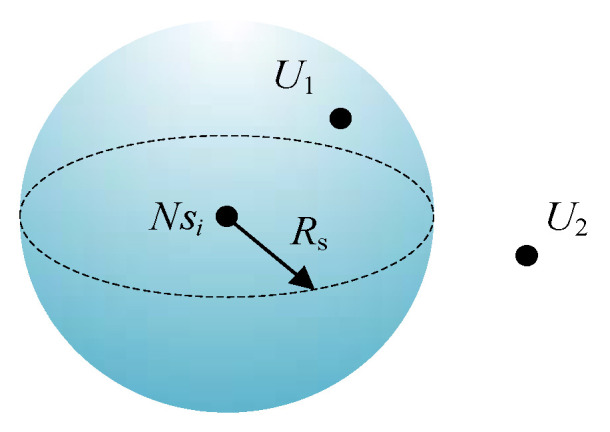
Coverage model.

That is, in this coverage model, the perception space of node *Ns_i_* is a sphere whose center is **X**(*Ns_i_*) = (*x_i_,y_i_,z_i_*) and radius is *Rs* as Figure 2 shows. The pixel that is in the perception sphere like *U*_1_ is covered by *Ns_i_*; on the contrary, the pixel that is out the perception sphere like *U*_2_ cannot be covered by *Ns_i_*.

Let *Ns =* **{***Ns*_1_*,Ns*_2_*,Ns*_3_*,…Ns_N_*} represents the nodes set of monitoring area. In this 3D network model as Figure 1, each sensor coverage is considered independent, and thus Equation (2) is utilized as follow to acquire the probability that *U* is covered by *Ns*.

(2) $$P_{\mathrm{cov}}=1-\prod_{Ns_{i}\in Ns}\left(1-p_{\mathrm{cov}}(x,y,z,Ns_{i})\right)$$

### 3.3. Coverage Rate Calculation

For the Boolean perception model as Equation (1), if at least one node of *Ns* can perceive the pixel *U*, it can be considered that *U* is covered by sensor nodes set *Ns*. The coverage rate is defined as Equation (3), which is the ratio of the volume of the effective monitoring area formed by all sensor nodes to the volume of the entire monitoring area *M*.

(3) $$\eta =\frac{Volume(\underset{Ns_{i}\in Ns}{\cup }Q_{i}\cap M)}{Volume(M)}$$

where, *η* is the coverage rate, *Volume* (.) is the volume of area, and *Q_i_* is the monitoring area of sensor node *Ns_i_*. Due to the irregular shape of the effective monitoring area, it is a NP-hard problem to accurately calculate the volume of the effective monitoring area using Equation (3). In practice, the mesh-grid based approach is a feasible method to estimate the covered volume. Dividing the monitoring area *M* into *l* × *w* × *h* grids, the intersections or center point of these grids can be denoted as *U*(*x,y,z*). The method mentioned above can be used to determine whether *U* is covered by *Ns*. Then, all covered grids are identified and their volumes are summed up, which is regarded as the approximation of the effective covered area. High-resolution mesh grid is helpful for improving the calculation accuracy of the covered volume; however, it will cause too long calculation time. In order to improve computing efficiency, the random sampling method such as Monte Carlo method can be adopted in practice.

### 3.4. Connectivity Definition

In this system, the connectivity rate is defined as follows:

(4) $$Con=\frac{N_{\mathrm{connect}}}{N}$$

where *N*_connect_ represents the number of sensors connecting to sink node.

### 3.5. Coverage Utilization

Let the probability that the node *Ns_i_* is covered by the neighboring node *Ns_j_* overlap be as Equation (5):

(5) $$p_{j}=\frac{V(Ns_{i},Ns_{j})}{\frac{4}{3}\pi Rs^{3}}$$

where, *V*(*Ns_i_*, *Ns_j_*) is the volume of the overlapping area covered by two nodes. Then, the probability that *Ns_i_* is not covered by neighbor node *Ns_j_* is expressed as Equation (6):

(6) $${\tilde{p}}_{j}=1-p_{j}$$

The coverage utilization of *Ns_i_* is defined as the probability that it is not covered by all *m* neighbor nodes:

(7) $$\tilde{P}(Ns_{i})=\prod_{j=j_{1}}^{j_{m}}\left(1-p_{j}\right)$$

It reflects the ratio of the area volume of the node *Ns_i_* that does not overlap with its neighbors to the volume of its own perception space.

### 3.6. Growth Ring and Forward Subtree Root Nodes

According to this proposed growth-ring based deployment strategy, in the connected tree rooted with sink node, the *g*-th ring of subtree root nodes (SRNs) set is called the *g*-th growth ring (GR). 

When the nodes in *Root_g_* collects and transmits data during the network deployment phase, the collected messages are forwarded by one or multiple hops to the previous ring of SRNs. Thus, the nodes in *Root_g_* are called the forward subtree root nodes (FSRNs).

### 3.7. Basic Node Set

During the network deployment process, the node set whose dive depth has been calculated in the current GR is called the basic node set (BNS), which is denoted as *basenodes*. It is a reference benchmark for depth calculation of unprocessed nodes, and initially *basenodes =* {Sink}. With the deployment process, the current nodes whose dive depths have been calculated are added to the BNS dynamically.

### 3.8. Horizontal Distance, Expect Distance and Offset Distance

For any two nodes *Ns_i_* and *Ns_j_*_,_ the horizontal distance (HD) between them is defined as the distance between *Ns_i_* and *Ns_j_* when both are placed on the same horizontal plane (that is, *Ns_i_*.*Z* = *Ns_j_**.**Z*). Let HD between *Ns_i_* and *Ns_j_* be denoted as *d*(*Ns_i_*,*Ns_j_*).

The expect distance (ED) between nodes *Ns_i_* and *Ns_j_* is defined as the distance to guarantee the connectivity between *Ns_i_* and *Ns_j_* during depth adjustment. Let ED between *Ns_i_* and *Ns_j_* be denoted as *Edist*(*Ns_i_*,*Ns_j_*). Its maximum value is *Rc*_max_, and the minimum value is *d*(*Ns_i_*,*Ns_j_*). In the practical depth adjustment, its value takes any element value in the set {*Rc*_max_, *Rc*_max_-∆*r*, …., *d*(*Ns_i_*,*Ns_j_*)}, where ∆*r* is the step length.

The offset distance (OD) between nodes *Ns_i_* and *Ns_j_* is defined as the possible maximum offset distance between them in the vertical direction when guaranteeing connectivity between *Ns_i_* and *Ns_j._* Let OD between *Ns_i_* and *Ns_j_* be denoted as *Div**_Ns_***(*Ns_i_*,*Ns_j_*). The relationship between these three distances is shown as Equation (8):

(8) $$Div_{Ns}(Ns_{i},Ns_{j})=\sqrt{Edist^{2}(Ns_{i},Ns_{j})-d^{2}(Ns_{i},Ns_{j})}$$

### 3.9. Network Reliability Definition

Let the numbers of nodes within *Rc*_max_ from the sink node be denoted as *N*_sin_. Let the average number of neighbor nodes for all sensors be denoted as *N*_avg._ *N*_sin_ and *N*_avg_ are calculated as Equation (9) and Equation (10), respectively:

(9) $$N_{\sin}={N|}_{dist(.,\mathrm{Sin}k)<Rc_{\max}}$$

(10) $$N_{\mathrm{avg}}=\frac{1}{N}\cdot \sum_{i=1}^{N}{N_{i}|}_{dist(.,Ns_{i})<Rc_{\max}}$$

where ${N|}_{dist(.,\mathrm{Sin}k)<Rc_{\max}}$ is the numbers of nodes within *Rc*_max_ from the sink node, and ${N_{i}|}_{dist(.,Ns_{i})<Rc_{\max}}$ is the numbers of nodes within *Rc*_max_ from node *Ns_i_*, i.e., the degree of *Ns_i_*.

The high values of these indicators mean that some other optional transmission paths can be selected to maintain the network connectivity. Therefore, the higher these two indicators are, the greater the reliability of the network is.

### 3.10. Deployment Energy Consumption

The mobile energy consumption of nodes moving from the water surface to a specified location represents the main energy consumption during self-deployment. To simplify calculations, the energy consumption for network deployment is expressed as follows:

(11) $$W=\frac{D}{v}\cdot p$$

where, *D* is the total distance that all nodes move during the deployment process, and *v* is the moving speed of nodes, and *p* is power. 

### 3.11. Problem Definition

The problem of this paper is defined as follows:

*N* sensors are dropped to water surface of monitoring area initially and a 2D connective network is formed. What is the optimal depth of each sensor so that the coverage of network reaches the maximum under the condition of energy consumption balance and guarantees connectivity to surface station?

## 4. Description of the Proposed Algorithm

### 4.1. The Whole Strategy for the Proposed Algorithm

Considering the sink node as the root node, the nodes that are scattered randomly on the water surface are organized as a tree structure. Then the depths of all nodes are computed iteratively with the goal of the maximized coverage utilization and energy balance. Finally, all the nodes dive to the new position once according to the computed depths. The whole strategy has three phases as follows:

(1) The 1st GR is determined. The 1st ring of connected tree is constructed rooted with sink node and the depths of the nodes within the 1st GR are computed.

The sink node broadcasts a message to the other nodes to identify its one-hop nodes with a variable communication radius *Rb*. The set of all these one-hop nodes which are identified by replying to this message is regarded as the subtree nodes attached to sink node and denoted as *S*_1_. The nodes in *S*_1_ are sorted by the distance to sink node from far and near. Selecting from these sorted nodes in *S*_1_, a SRNs set is made with a certain probability based on the principle that the distance between each other is roughly uniform. This set is called the 1st GR and denoted as *Root*_1_. On the contrary, the other nodes in *S*_1_ are regarded as common child nodes of sink node. *S*_1_ is called the nodes within the 1st GR (1st NGR). So far, a connected tree is constructed rooted with sink node. Meanwhile, the optimized depths of the nodes in *S*_1_ are computed with the goal of maximizing coverage utilization and energy balance.

(2) Ring by ring, each ring of connected tree is constructed rooted with the previous GR and the depths of all nodes are calculated.

Each node in *Root*_1_ broadcasts a message to the other nodes to identify its next hop nodes with the increased broadcast radius *Rb* which is larger than that of the previous iteration. The set of these next-hop nodes which are identified by replying to this message is regarded as the subtree nodes attached to each respective node in *Root*_1_ and all these next-hop nodes are called the nodes within the 2nd GR (2nd NGR) denoted as *S*_2_. Then, these nodes in *S*_2_ are sorted by the distance to sink from far and near so that the SRNs set of the second ring is determined using the similar method with the first phase. The SRNs set of the second ring is denoted with *Root*_2_ and called the 2nd GR. On the contrary, the other nodes in *S*_2_ are regarded as common child nodes of their corresponding subtree. Starting from each node in *Root*_1_, the optimized depths of the nodes in *S*_2_ are computed. So far, a series of connected trees are constructed rooted with the corresponding nodes in *Root*_1_. The above process is performed iteratively until all nodes are added into their corresponding subtree and the depths of all the nodes are computed.

(3) Sink node distributes the depths to the nodes so that all the nodes dive to the new position once.

**Figure 3:**
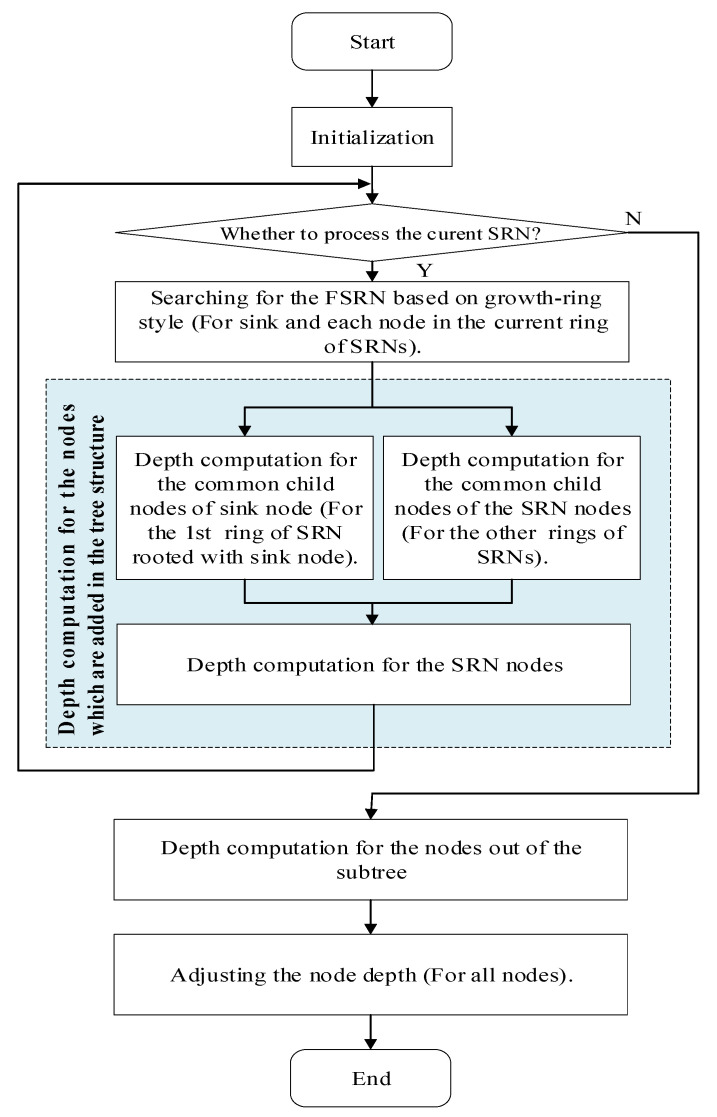
The flow chart of the whole strategy.

This strategy finally forms a growth ring like network layout. The radius of the growth ring increases with the distance to sink node increasing. The flow chart of the whole strategy is shown in Figure 3.

**Figure 4:**
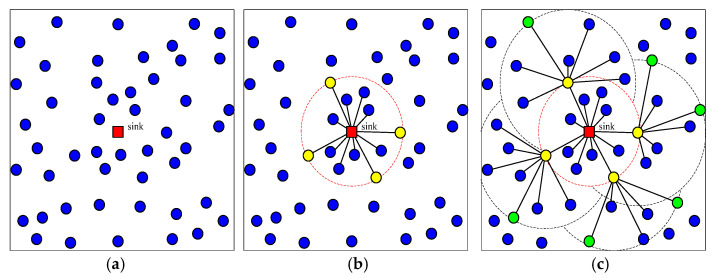
The overall strategy of autonomous depth-adjustment deployment method in this paper. (**a**) Initialization. (**b**) The first growth ring. (**c**) The second growth ring.

Figure 4 shows an illustration of this deployment strategy. The sensor nodes are dropped randomly to the water surface of the monitoring area initially as shown in Figure 4a, where the red node denotes the sink node. The connected tree which is rooted with sink node in the first phase of the strategy is shown in Figure 4b, where the yellow nodes denote *Root*_1_, i.e., the 1st GR. The 2nd NGRs and the connected trees which are rooted with the corresponding the nodes in *Root*_1_ are shown in Figure 4c, where the green nodes denote *Root*_2_, i.e., the 2nd GR.

According to this proposed deployment strategy, the connected trees in which the nodes are farther from the sink node have a larger communication distance, thus the node layout is sparse. On the contrary, the connected trees in which the nodes are nearer to the sink node have a smaller communication distance and the node layout is dense. Therefore, there are more nodes in hot-spots area near the sink node, so that network reliability is improved and network transmission energy consumption is balanced. Moreover, the network coverage is improved and the connectivity is guaranteed with the process of optimizing the nodes depth in the connected trees.

### 4.2. Initialization

*N* sensor nodes are dropped randomly to the water surface with sink node as the central node. Let the coordinate of the sink node in 3D space be (*Length*/2, *Width*/2, 0), and its *id* be 0. The relative coordinates of the other nodes are denoted as **X**(*Ns_i_*) = (*x_i_*, *y_i_*, 0). Each node is initialized with the following attributes:

< *id* = *i*, *tree* = 0, *leaf* = 1, *father* = *N* + 1, *root* = Inf, *processe*d = 0 >

where, *id* is the unique identification of each node, *tree* indicates that whether the node joins the tree structure, *father* is the *id* of the parent node and its initial value is set with *N* + 1, *root* is the root node of the tree to which the node is attached and its initial value is set with Inf, *processed* indicates whether the current node has calculated the diving depth. If *leaf* = 1, it means that the node is a leaf node of tree. 

Let the number of GR *g* = 0, the *g*-th ring of SRNs set *Root_g_* = Φ. The distances between each node and the sink node are calculated and denoted as *dist*(*Ns_i_*, Sink). An ordered node set is then constructed according to these distances from largest to smallest using Equation (12):

(12) $$distnodes=\{Ns_{j}|j=1,2,\ldots ,N,\;dist(Ns_{j},\mathrm{Sin}k)>dist(Ns_{j+1},\mathrm{Sin}k)\}$$

### 4.3. Searching for the FSRN Based on Growth Ring Style

The algorithm for searching the FSRN based on ring style is described by Algorithm 1 as follows.
**Algorithm 1** The algorithm for searching the FSRN based on growth ring style.
**Step 1**. The sink node broadcasts a message including < *id* = 0, *g* = 0 > to the other nodes with the broadcasting radius *Rb*, which is calculated by Equation (13):(13)$$Rb=\min(Rb_{1},Rb_{2})$$$$Rb_{1}=\alpha \cdot Rs+(Rc_{\max}-\alpha \cdot Rs)\cdot g\cdot \beta ,Rb_{2}=\sqrt{Rc_{\max}^{2}-Rs^{2}}$$where, *α*, *β* are parameters. The broadcast radius increases with the growth ring number *g* increasing, which makes the radial distance between GRs increasing gradually.Each node *Ns_i_* that receives the broadcast message responses to the sink node with a message including < *id* = *i*, *dist*(*Ns_i_*,Sink) >. Then, the sink node sets *Ns_i_*.*tree* = 1, *Ns_i_*.root = 0, that is, *Ns_i_* is regarded as the subtree node of the sink node.**Step 2**. According to the order of nodes in *distnodes*, each node *Ns_i_* that *Ns_i_*.*tree* = 1and *Ns_i_*. *processed* = 0 is processed and the SRNs set *Root_g_* is constructed as follows.If *Root_g_* = Φ: A random number is generated as *r* = *rand*(). If *r* > *th*, where *th* is a threshold, the farthest node *Ns_i_* in *distnodes* that *Ns_i_*.*tree* = 1 and *Ns_i_*. *processed* = 0 is immediately added into *Root_g_.* Then let *Ns_i_*.*father* be set with the *id* of the root of the subtree to which it belongs. Let *Ns_i_*. leaf = 0, that is, the node *Ns_i_* is a non-leaf node; otherwise, the next far node in *distnodes* that meets the candidate conditions continues to be considered.If *Root_g_* ≠ Φ: The node *Ns_i_* is determined whether to join in the subtree according to the following method. Let *Ns_j_* be an arbitrary node in *Root_g_.* If both *Ns_i_* and *Ns_j_* satisfy the condition of Equation (14):(14)$$dist(Ns_{i},Ns_{j})\geq \alpha \cdot Rs+\gamma \cdot g$$where, *α*, *γ* are parameters and the value of *α* is same with that of Equation (13), *Ns_i_* will be added into the *Root_g_*. The distribution of these nodes in *Root_g_* acquired with this method is roughly uniform. Furthermore, this method can reduce the coverage overlap between subtree nodes and it is also good for improving the node utilization ratio.**Step 3**. The depths of each SRN and its children are calculated and a connected tree is constructed with the method mentioned in Section 4.4. Then, these children nodes send their collected message to their corresponding parents, i.e., the FSRNs. The parent nodes aggregate these messages from the children in the subtree and forward the messages to FSRNs. The above procedure is performed repeatedly until sink node receives those collected messages.**Step 4**. Each node in *Root_g_* is denoted as FSRNs for the next GR. *g* ← *g +* 1. Then, these FSRNs broadcast messages including < *id* = *i*, *g* > to the next-hop nodes synchronously with the broadcasting radius *Rb*, which is calculated as Equation (13). Each node *Ns_i_* that receives the broadcast message and *Ns_i_*.*tree =* 0 responses to the nearest FSRN node *Ns_j_* with a message including < *id* = *i >*. Then, set *Ns_i_*.*tree* = 1 and *Ns_i_*.*root = Ns_j._id.***Step 5**. Go to step 2 and execute step2–4 repeatedly until *g > δ* or all nodes are added to the tree structures, where *δ* is the maximum of GR and it can be calculated as Equation (15):(15)$$\delta =\left\lceil \frac{\max(\frac{Length}{2},\frac{Width}{2})}{\alpha \cdot Rs}\right\rceil +1$$

### 4.4. Depth Computation for the Nodes Which Are Added in the Tree Structure

#### 4.4.1. Depth Computation for the Common Child Nodes of Sink Node

For each common child nodes *Ns_i_* of sink node, its depth is calculated with the goal of maximizing coverage utilization and energy balance, meanwhile, and guaranteeing connectivity to the sink node. This paper firstly uses each node in the BNS as a benchmark to calculate the local optimal dive depth. Then, with the goal of improving coverage and energy balance, find the global optimal dive depth from all local optimal dive depths:

##### Calculation of local optimal dive depth based on basic node

**Figure 5:**
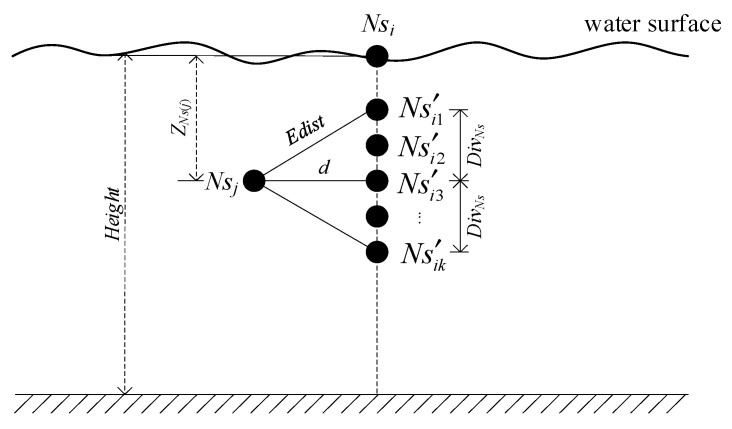
The set of dive candidate positions for *Ns_i_*.

Let the current BNS be *basenodes*, and the common child node to be calculated depth be *Ns_i_*. For each node *Ns_j_* in *basenodes*, if *d*(*Ns_i_*,*Ns_j_*) < *Rc*_max_, where *d*(*Ns_i_*,*Ns_j_*) is the HD between *Ns_i_* and *Ns_j_*, *Ns_j_* is regarded as the basic node to calculate the optimal depth of *Ns_i_*. Otherwise, the other nodes in *basenodes* are selected to determine the basic node under the condition of *d*(*Ns_i_*,*Ns_j_*) < *Rc*_max_. Let the basic node of *Ns_i_* be selected as *Ns_j_.* Then, according to the definition of Section 3.8, the dive candidate positions that *Ns_i_* may choose are the positions of the discrete points whose depth is in [*Z**_Ns_***_(***j***)_ − *Div**_Ns_***, *Z**_Ns_***_(***j***)_ + *Div**_Ns_***], as Figure 5 showing, where *Z**_Ns_***_(***j***)_ is the calculated depth of the basic node *Ns_j_*.

Let the dive candidate position points set of *Ns_i_* based on basic node *Ns_j_* be $Candidate(Ns_{i})=\{N{s^{\prime }}_{i1},N{s^{\prime }}_{i2},\ldots ,N{s^{\prime }}_{ik}\}$. If the coverage area of $N{s^{\prime }}_{ik}$ is above the water surface or below the water bottom, that is, $N{s^{\prime }}_{ik}.Z<\frac{Rs}{2}$ or $N{s^{\prime }}_{ik}.Z>Height-\frac{Rs}{2}$, the coverage area is invalid. Hence the depth of the candidate position of $N{s^{\prime }}_{ik}$ is revised as Equation (16).

(16) $$N{s^{\prime }}_{ik}.Z=Rs\cdot random(0.5,1),\;\mathrm{if}\;N{s^{\prime }}_{ik}.Z<\frac{Rs}{2}$$

$$N{s^{\prime }}_{ik}.Z=Height-Rs\cdot random(0.5,1),\;\mathrm{if}\;N{s^{\prime }}_{ik}.Z>Height-\frac{Rs}{2}$$

where, *random*(*a*,*b*) represents a random number in [*a*,*b*], and *Height* is the depth of water bottom as Figure 5 showing. Then, the current node *Ns_i_* is virtually placed at each candidate position and its overlap area with other nodes whose depth has been calculated (*processed* = 1) is calculated as Equation (17):

(17) $$V(Ns_{i},Ns_{j})=\frac{4}{3}\pi Rs^{3}-\pi \cdot dist(Ns_{i},Ns_{j})\cdot (Rs^{2}-\frac{dist{\left(Ns_{i},Ns_{j}\right)}^{2}}{12})$$

where *dist*(*Ns_i_, Ns_j_*) is the Euclidean distance between *Ns_i_* and *Ns_j_*. The smaller the overlap area is, the higher the coverage utilization of the node is at this position. Because the distance between *Ns_i_* and *Ns_j_* is smaller than 2*Rs*, there must be an overlap area between them.

Considering *Ns_j_* as the basic node, the local optimal dive candidate position of *Ns_i_* is the position where the coverage utilization is the largest according to the definition in Section 3.5. It can be represented by Equation (18):

(18) $$Ns_{i}=\underset{N{s^{\prime }}_{i}\in Candidate(Ns_{i})}{\arg\max}\tilde{P}(N{s^{\prime }}_{i})$$

**Figure 6:**
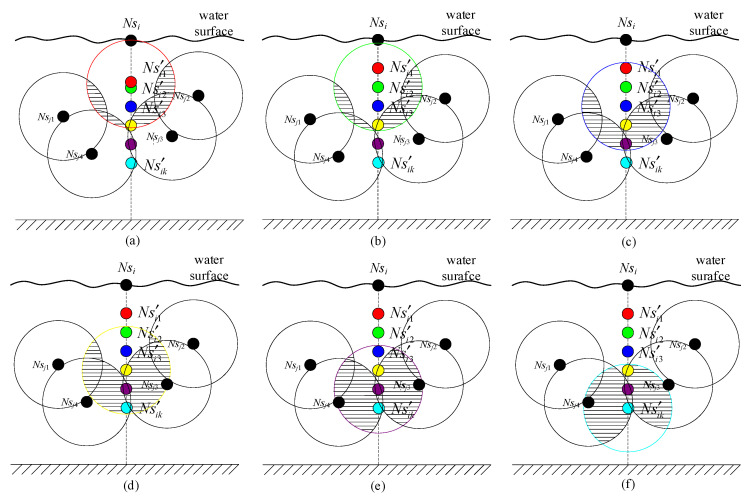
The optimal depth computation for *Ns_i_* based on *Ns_j._* (**a**) The overlap for $N{s^{\prime }}_{i1}$. (**b**) The overlap for $N{s^{\prime }}_{i2}$.(**c**) The overlap for $N{s^{\prime }}_{i3}$. (**d**) The overlap for $N{s^{\prime }}_{i4}$. (**e**) The overlap for $N{s^{\prime }}_{i5}$. (**f**) The overlap for $N{s^{\prime }}_{i6}$.

Figure 6 shows the calculation process of the optimal dive depth based on *Ns_j_* using Equation (18). When the dive candidate positions of *Ns_i_* are selected with $Candidate(Ns_{i})=\{N{s^{\prime }}_{i1},N{s^{\prime }}_{i2},N{s^{\prime }}_{i3},N{s^{\prime }}_{i4},N{s^{\prime }}_{i5},N{s^{\prime }}_{i6}\}$ respectively, the volumes for the overlap areas between $N{s^{\prime }}_{ik}$(*k* = 1, 2, …, 6) and *Ns_j_*_1_, *Ns_j_*_2_, *Ns_j_*_3_, *Ns_j_*_4_, whose depths have been calculated, are shown as Figure 6a–f, where the shadow areas indicate the overlap areas. Assuming Figure 6b has the largest coverage utilization in all candidate positions of *Ns_i_*, $N{s^{\prime }}_{i2}$ is then the local optimal dive candidate positions for *Ns_i_* based on *Ns_j_*.

##### The Model Based on Coverage Utilization and Energy Balance

In order to improve the coverage rate while guaranteeing the energy balance in the network deployment, the model based on coverage utilization and energy balance (MBCUEB) is constructed when calculating the global optimal dive depth. This model is described as Equation (19):

(19) $$f(Ns_{i})=a\cdot \tilde{P}(Ns_{i})+b\cdot \frac{dist_{\max}-dist(Ns_{i},\mathrm{Sin}k)}{dist_{\max}}$$

where, $\tilde{P}(Ns_{i})$ is the coverage utilization of *Ns_i_* defined as Equation (7), *dist*(*Ns_i_,* Sink) is the Euclidean distance between *Ns_i_* and sink node, and *dist*_max_ is the maximum distance of all nodes from the sink node. The smaller the distance between the node *Ns_i_* and the sink node is, the larger the value of $\frac{dist_{\max}-dist(Ns_{i},\mathrm{Sin}k)}{dist_{\max}}$ will be, on the contrary, the large distance between *Ns_i_* and sink node means the small value of this ratio. Thus, this ratio reflects the characteristics of node distribution when energy is balanced. The parameters *a*, *b* are the weights, and *a* + *b* = 1. Hence, *f*(*Ns_i_*) reflects the comprehensive performance of *Ns_i_* on coverage utilization and energy balance.

##### The Global Optimization Frame for Depth-Adjustment

Based on the analysis above, the global optimization model of depth-adjustment for node *Ns_i_* can be set up as Equation (20):

(20) $$\left\{ \begin{array}{l} J=\max\;f(Ns_{i})\\ s.t.\\ C1:\;Ns_{i}\in \left\{N{s^{\prime }}_{iq}|N{s^{\prime }}_{iq}=\underset{N{s^{\prime }}_{i}\in Candidate(Ns_{i})}{\mathrm{argmax}}\tilde{P}(N{s^{\prime }}_{i}),q=1,2,\ldots \right\}\\ C2:\;\left(x_{i},y_{i},z_{i}\right)\in M,\;Ns_{i}\in Ns\\ C3:\;Z_{Ns}{}_{\left(j\right)}-Div_{Ns}\leq N{s^{\prime }}_{i}.Z\leq Z_{Ns}{}_{\left(j\right)}+Div_{Ns} \end{array}\right.$$

This is a constrained optimization problem. The constraint C1 indicates that the global optimal position *Ns_i_* is selected from all local optimal positions based on each basic node. The constraint C2 means that *Ns_i_* must always be within the monitoring area. The constraint C3 means that the depth of candidate node should guarantee the connectivity with its basic node.

According to this optimization model, the global optimal dive depth of *Ns_i_* is solved as follows. Firstly, each node in *basenodes* is utilized as a basic node to calculate the local optimal dive candidate position of *Ns_i_* adopting the previous section method. Then, for each candidate position of *Ns_i_*, the MBCUEB defined as Equation (19) is adopted to calculate the corresponding *f*(.) value. The position with the maximum value of *f*(.) is the global optimal position of *Ns_i_*.

**Figure 7:**
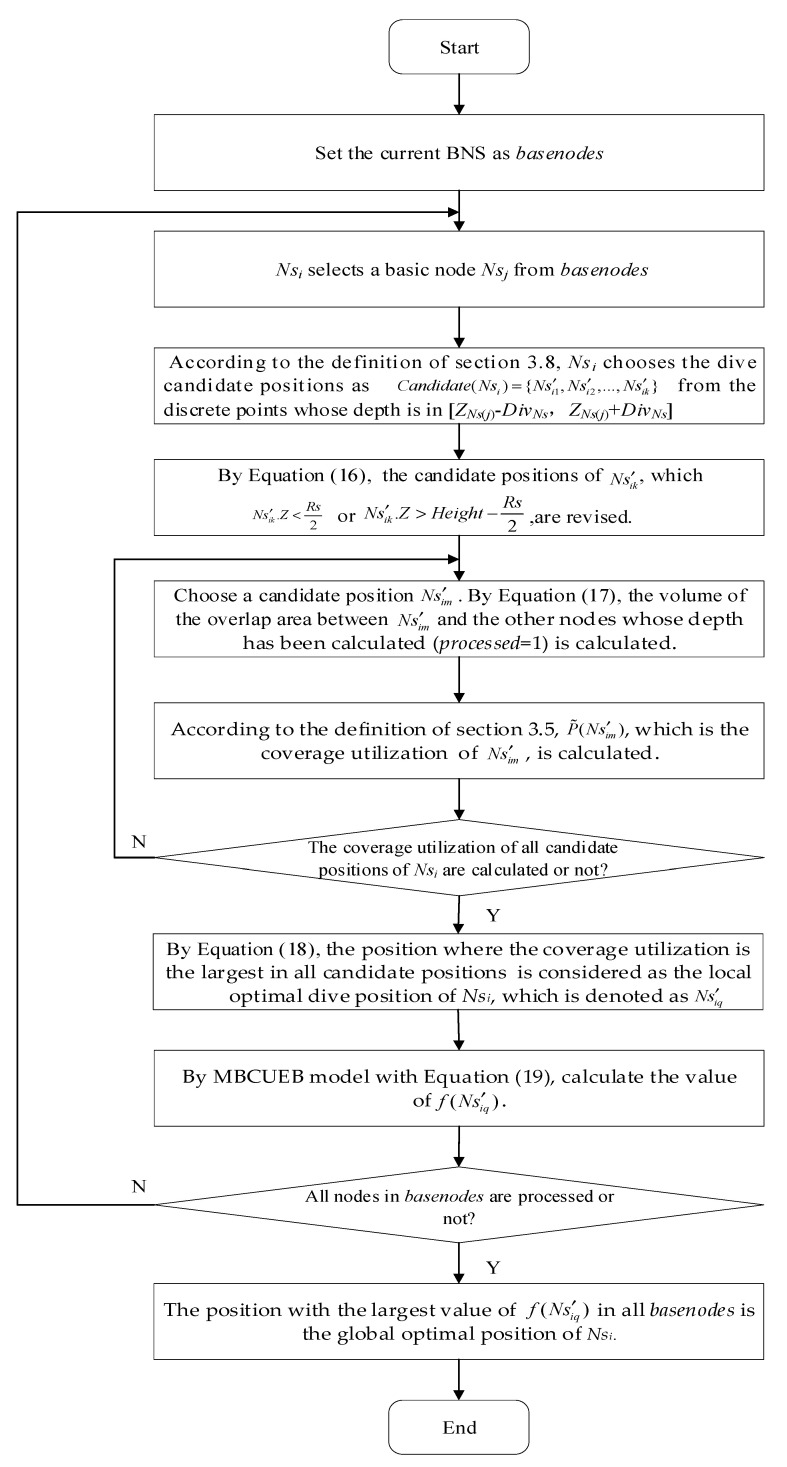
Flow chart of calculation optimal dive depth with *Ns_i_* as an example.

The flow chart of calculation of local optimal dive depth based on basic node and global optimal dive depth is shown in Figure 7 with *Ns_i_* as an example.

##### Description of the Algorithm

The algorithm of depth computation for the common child nodes of sink node is described by Algorithm 2 as follows.
**Algorithm 2** Depth computation for the common child nodes of sink node
**Step 1**. Let *basenodes* be the BNS. Initially, set *basenodes =* {Sink}.**Step 2**. For each common node *Ns_i_*, the method mentioned in Section “Calculation of local optimal dive depth based on basic node” above is adopted to select the basic node *Ns_j_* and calculate the local optimal dive depth based on this basic node *Ns_j_*.**Step 3**. Taking each node in *basenodes* as the basic node, calculate the corresponding local optimal dive depth. Then the method mentioned in Section “The model based on coverage utilization and energy balance” and Section “The global optimization frame for depth-adjustment” above is adopted to calculate global optimal dive depth of *Ns_i_*. The current basic node corresponding to the global optimal depth is the parent node of *Ns_i_*. Set *Ns_i_*.father with the *id* of the basic node, *Ns_i_.processed* = 1. Add *Ns_i_* to *basenodes.***Step 4**. Go to step 2 and execute step2–3 repeatedly until the depths of all common children of sink node are calculated.

#### 4.4.2. Depth Computation for the SRN Nodes

For the SRN nodes of *Root_g_*, if *Ns_i_*. *processed* = 0, its depth is calculated as Equation (21).

*NS_i_*.*Z* = *Rs* (21)

#### 4.4.3. Depth Computation for the Common Child Nodes of the SRN Nodes

The depth computation for the common child nodes of the SRN nodes is similar with the method mentioned above using Equations (16)–(20). Meanwhile, the connection paths to the sink node are guaranteed.

### 4.5. Depth Computation for the Nodes out of the Subtree

For some nodes that are not yet added in the connective tree structure through the above process, especially on the border of the monitoring area, the process is performed as Algorithm 3 until all nodes are added in the tree structures.
**Algorithm 3** Depth computation for the nodes out of the subtree**Step 1**. Select a node *Ns_j_* from the nodes whose depth has been calculated under the condition that *d*(*Ns_i_*,*Ns_j_*) < *Rc*_max_, where *d*(*Ns_i_*,*Ns_j_*) is the HD between *Ns_i_* and *Ns_j_*. Then, *Ns_j_* is considered as the neighbor node of *Ns_i_* on the horizontal plane.**Step 2**. *Ns_i_* is added to the tree structure rooted with *Ns_j_*. If *d*(*Ns_i_*, *Ns_j_*) ≥ α* *Rs*, the depth of *Ns_i_* is set the same with that of *Ns_j_*. Otherwise, the local optimal dive depth of *Ns_i_* is calculated using Equations (16)–(18) considering *Ns_j_* as the basic node.When selecting the node *Ns_j_*, its degree (i.e., the number of its children) should be inspected. If the degree of *Ns_j_* is greater than *μ*, where *μ* is a non-negative integer threshold, it means that *Ns_j_* is overloaded for network transmission. In order to balance the energy consumption, another node is selected from the nodes whose dive depth has been calculated.**Step 3**. The above process is performed repeatedly until all nodes are added in the tree structures and their depths are calculated.

### 4.6. Adjusting the Node Depth

This process is performed based on the connected tree structure. Firstly, the sink node broadcasts the depths of all nodes to the first ring of SRNs and its common child nodes. Then, the first ring of SRNs forwards these depths message to the second ring of SRNs. This process is performed in turn until all SRNs received the depths messages. After that, each ring of SRNs broadcast the depths to their common child nodes until the leaf nodes. Finally, all the nodes dive to the new position once.

### 4.7. Algorithm Flow

**Figure 8:**
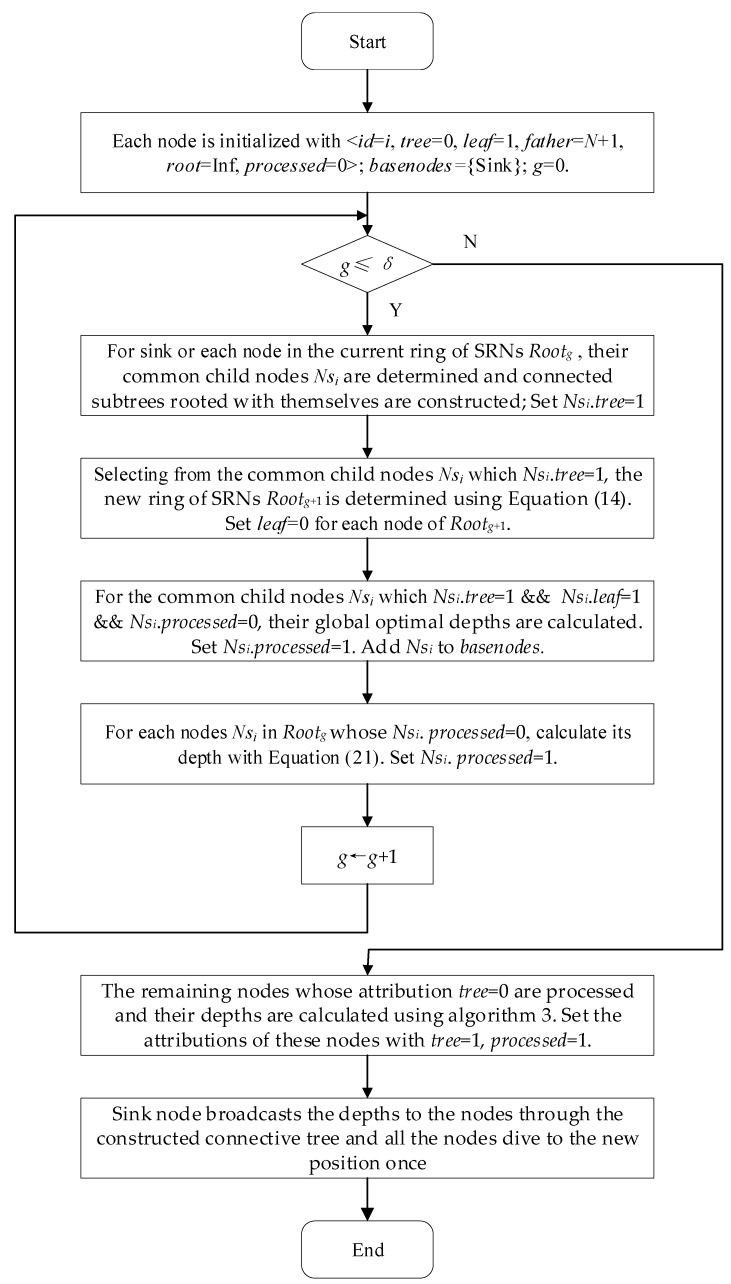
Flow chart of GRSUNDSOA.

The flow chart of GRSUNDSOA is shown in Figure 8. Initially, the network is initialized which is rooted with the sink node using the method mentioned in Section 4.2. Then, a connected subtree is constructed regarding the sink node as the root node and every ring of SRNs (i.e., GR) constructs the connected subtrees rooted with itself using the method in Section 4.3. The global optimal depths of the common child nodes of sink node and SRNs are calculated using the method in Section 4.4.1 and Section 4.4.3. The depths of SRNs are calculated using the method in Section 4.4.2. This procedure is performed repeatedly until *g > δ* or all nodes are added to the tree structures and their depths are calculated. Next, the remaining nodes that have not been added to the tree structure are processed and their depths are calculated using the method mentioned in Section 4.5. Finally, all nodes are adjusted to the positions of the calculated dive depth using the method of Section 4.6.

In practice, the mobility of node in the underwater environment can result in the change of horizontal position due to waves and water current. These factors can decrease the network performance. This problem is addressed with the method described in [29] in this paper.

## 5. Algorithm Simulation and Analysis

### 5.1. Theory Analysis

#### 5.1.1. Feasibility Analysis

In the environment of 3D underwater, the superposition of all nodes covering perception sphere is an irregular shape. Thus, it is a NP-hard problem to use Equation (3) to accurately calculate the coverage volume of node set *Ns*. This proposed algorithm does not require the underwater coordinate position information of the node calculated by GPS; moreover, it only requires the relative coordinate positions of nodes on the water surface. This is feasible for 3D UWSN deployment.

#### 5.1.2. Connectivity Analysis

The sink node or SRN broadcasts a message to the other nodes to identify their one-hop nodes with a variable communication radius *Rb*, where $Rb<\sqrt{Rc_{\max}^{2}-Rs^{2}}$, thus nodes belonging to sink or SRN are connected with each other in horizontal plane. Moreover, as mentioned in Section 3.8, when calculating OD, the maximum ED is taken with *Rc*_max_, thus the node whose depth is to be calculated and its basic node are always connected as known from Equation (8). Therefore, the process of depth-adjustment guarantees that the network is always connected. 

#### 5.1.3. Energy Balance Analysis

According to this proposed deployment strategy, the deployed SRNs far from sink node have a larger communication radius, thus the corresponding sub-trees far from sink node have a larger communication distance and they have a large vertical adjustment space. Therefore, the node density in this area far from sink node is small. On the contrary, the sub-trees near the sink node have smaller communication distances and they have a small vertical adjustment space. Therefore, the node density and the number of leaf nodes in this area near the sink node are large. The node distribution deployed with GRSUNDSOA is non-uniform. Moreover, when calculating the dive depth of nodes, it not only considers the local optimal dive depth based on basic nodes with the goal of maximizing the coverage utilization, but also considers the global optimal dive depth from all local optimal dive depths with the goal of improving coverage utilization and energy balance. Therefore, according to this proposed deployment strategy, there are more nodes in hot-spots area near sink node and the node density is different in different under water areas. So that network transmission energy consumption is balanced and it can avoid the energy hole problem effectively.

#### 5.1.4. Complexity Analysis

##### Message Complexity

The algorithm mainly includes the following aspects of message transmission when deploying the network. Initially, the sink node broadcasts a message to the other nodes with a variable communication radius and the nodes which receive this message reply to sink. Ring by ring, the nodes in each GR broadcast a message to the other nodes with a variable communication radius and the nodes receiving this message send a response message to the corresponding node in GR. Finally, the sink node sends the calculated depths to each node through the connected tree. Therefore, the message complexity of each node is *O*(1). For UWSNs composed of *n* nodes, the total message complexity is *O*(*n*).

##### Time Complexity

For UWSNs composed of *n* nodes, the time consumption of GRSUNDSOA is mainly in the calculation of the dive depths. The proposed algorithm needs to calculate the depths of *n* nodes one by one. For the depth calculation of each node, let the maximum number of nodes in BNS be *d*, and then the local optimal dive depth of each basic node is to be calculated. For the local optimal depth calculation of each basic node, it needs to calculate the overlap area between each candidate best position and the nodes whose depths have been calculated. Let the maximum number of candidate best position be *k*. Therefore, the time complexity of GRSUNDSOA is *O*(*ndk*).

### 5.2. Simulation and Experimental Analysis

#### 5.2.1. Simulation Scenario and Parameter Settings

In order to verify the effectiveness of GRSUNDSOA, the proposed algorithms are implemented and the nodes deployment process are simulated by a MatLab program. The simulation experiments are performed on a computer equipped with an Intel Core i7-8550U 1.8 GHz CPU and 8.0 G RAM. GRSUNDSOA is compared with four approaches— Cacc [28], CDA [26,27], VODA [29]—and the random based deployment approach in this simulation. To fully verify the algorithms performance, each simulation is conducted 20 times. The monitoring area is set to 200 m × 200 m × 500 m. Some parameter settings used in the simulations are shown in Table 1. The definitions of these parameters are shown in glossary section.

#### 5.2.2. Analysis of the Effect of Parameter α on the Algorithm

In this experiment, the number of nodes *N* is fixed to 120 and *α* value is varied from 1.4 to 1.8 with the step length 0.1. The effects of *α* on three indicators, namely, coverage rate, average node degree *N*_avg_ and the sink node degree *N*_sin_ in *Rc*_max_ are compared and analyzed.

**Figure 9:**
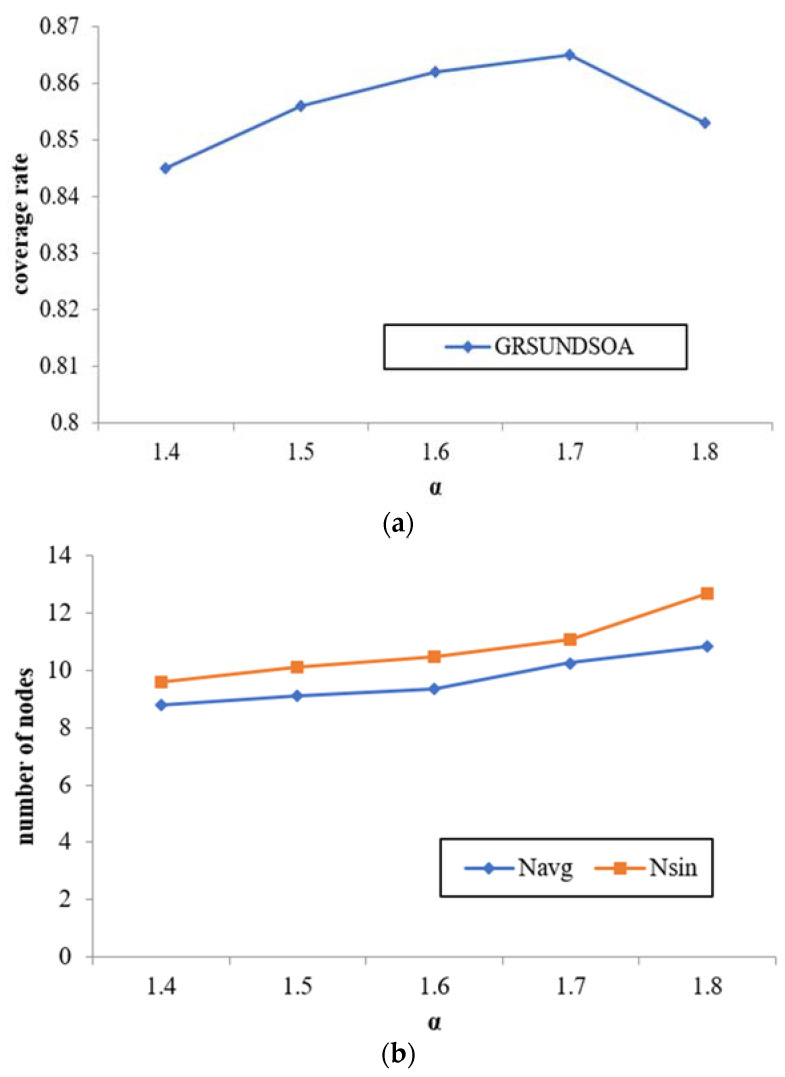
Effect of parameter *α* value on network performance. (**a**) The effects of *α* on coverage rate. (**b**) The effects of *α* on node degree.

As shown from Figure 9, with the increase of *α* value, both the broadcast radius and the distances between SRNs increase. The number of SRNs in the 1st GR increases during the execution of GRSUNDSOA, that is, the number of constructed sub-trees increases, and meanwhile, the number of the nodes in each sub-tree also increases. Therefore, the average node degree and the sink node degree in *Rc*_max_ increase obviously, on the contrary, the change in coverage rate is relatively slight.

In the following experiments, *α* value is fixed to 1.4, and the number of nodes *N* varies from 80 to 160.

#### 5.2.3. Coverage Rate Comparison

**Figure 10:**
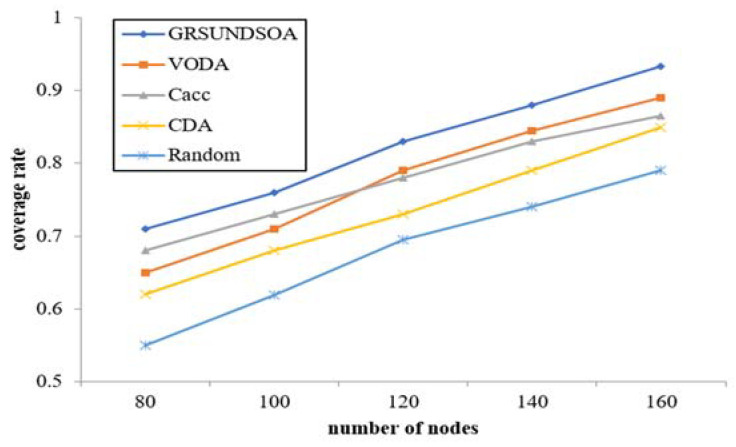
Coverage rate analysis for varying number of nodes.

The coverage rate comparison among five algorithms with various number of nodes is shown in Figure 10.

This figure shows that the coverage rate of five algorithms increases with the increasing of the number of nodes, but the trend slows down. GRSUNDSOA shows better coverage rate than that of the other four deployment approaches with the same number of nodes. Compared with the random based deployment approach, the coverage rate of GRSUNDSOA is increased by about 15%–30%. The coverage rate of GRSUNDSOA is also significantly improved compared to Cacc, CDA, and VODA algorithms. When selecting the dive position of nodes, CDA only compares the coverage redundancy rates on the position where the distance between the node and each of its basic nodes is *Rc*_max_ but does not consider the situation where the distance between them is less than *Rc*_max_. Similarly, VODA only calculates the dive depth on the position with a special ratio of the communication radius to the sensing radius. The dive positions calculated by both methods are often the local optimal dive positions rather than the global optimal positions. Different from CDA and VODA, GRSUNDSOA not only searches the local optimal dive position based on a certain basic node, but also searches the global optimal dive position based on the basic nodes whose depths have been calculated in the previous process. Therefore, the coverage rate of GRSUNDSOA is improved greatly.

#### 5.2.4. Connectivity Comparison

For GRSUNDSOA, the maximum communication radius *Rc*_max_ is always considered in the process of constructing connected tree structure, which guarantees the connectivity of the generated network topology. Therefore, like the Cacc, CDA, VODA algorithms, GRSUNDSOA guarantees the connection path from the sensors to sink node, that is, *Con* = 100% calculated with Equation (4). However, that random based deployment approach cannot guarantee the network connectivity.

#### 5.2.5. Analysis of the Average Node Degree, the node degree near Sink Node and Energy Balance

The indicators *N*_avg_ and *N*_sin_ mentioned in Section 3.9 reflect not only the reliability but also the load balance performance of network. Because the load of the nodes near the sink node is always greater, it is helpful for increasing routing and reducing energy consumption that increases the number of nodes near the sink node. Therefore, it can effectively balance the load of nodes and prevent nodes failure from energy exhausting prematurely. Thus, it can extend the lifecycle of network.

**Figure 11:**
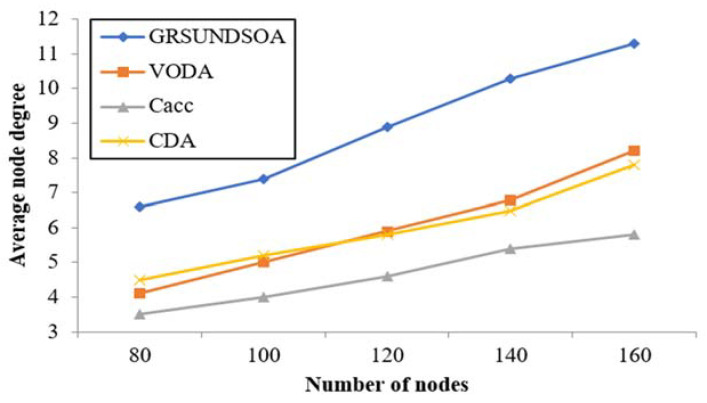
Comparison of the average node degree.

**Figure 12:**
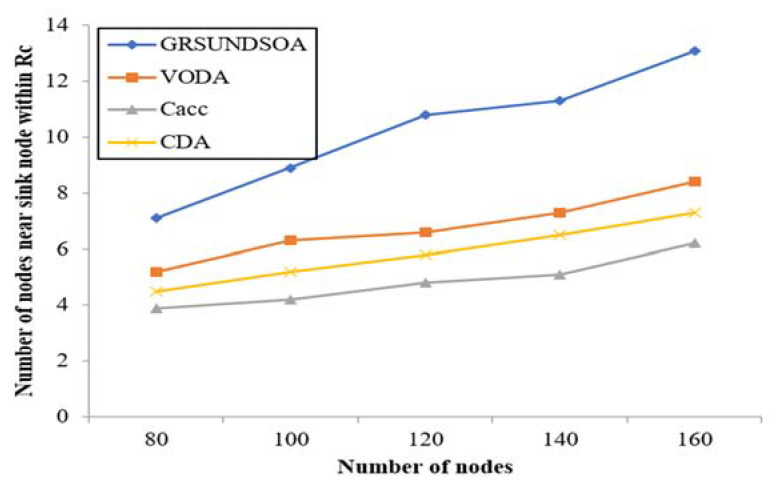
Comparison of the number of nodes near sink node within radius *Rc*_max_.

As shown in Figure 11 and Figure 12, the average node degree *N*_avg_ and the sink node degree *N*_sin_ in *Rc*_max_ of GRSUNDSOA are significantly higher than that of Cacc, CDA, and VODA for the varying number of nodes. It indicates that the network deployed by GRSUNDSOA can achieve better load balance between routing and higher reliability. As the broadcast radius of GRSUNDSOA increases gradually, the distances between SRNs increase gradually and the number of each ring of SRNs is roughly uniform. Therefore, the nodes distribution deployed by GRSUNDSOA is non-uniform. The nodes number near sink node is large and their distribution is dense. On the contrary, the nodes number far from sink node is small and their distribution is relative sparse. This conforms to the hot spot characteristics near the sink node and its energy consumption is more balanced. In addition, the proposed algorithm constructs the MBCUEB model and constrainted optimization model when calculating the global optimal dive depth. These models reflect the comprehensive performance of node on coverage utilization and energy balance. 

In a word, GRSUNDSOA can avoid the energy hole phenomenon effectively and extend the lifecycle of the network.

#### 5.2.6. Average Path Length

**Figure 13:**
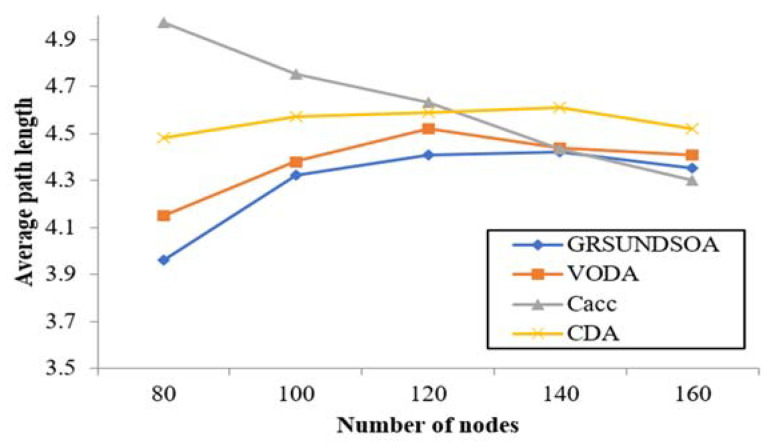
Comparison of average path length.

The average path length for each sensor to the sink node shows the average hops number for information transmission via nodes. In GRSUNDSOA, because the number of nodes near sink node is large, one hop distance is shorter. On the contrary, one hop distance is larger for the nodes far from the sink node especially on the border of monitoring area. Thus, as shown in Figure 13, with the number of nodes increasing, the change of average path length of GRSUNDSOA is slight and it is always lower than that of CDA and VODA.

#### 5.2.7. Deployment Energy Consumption

**Figure 14:**
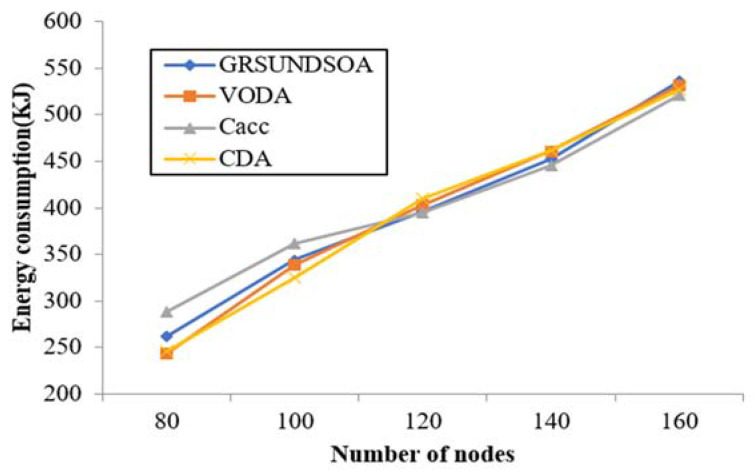
Comparison of energy consumption for network deployment.

To estimate the energy consumption of network deployment, the deployment energy consumption model is utilized as Equation (11), where the diving speed of nodes *v* is set at 2.4 m/min, and the power *p* is set with 0.6 W in this experiment. As shown in Figure 14, the energy consumption for deployment of GRSUNDSOA is not much different from that of the other three algorithms, CDA, Cacc, and VODA.

## 6. Conclusions

In this paper, a growth ring style uneven node depth-adjustment self-deployment optimization algorithm for UWSNs is proposed. Through a series of simulation experiments, the proposed GRSUNDSOA algorithm is compared with the CDA, Cacc and VODA algorithms in terms of several performance indicators such as coverage rate, connectivity, average node degree, average path length, energy balance, and deployment energy consumption. The simulation results with varying number of nodes indicate that GRSUNDSOA has better coverage, network reliability and energy balance performance than those of CDA, Cacc and VODA. It can avoid the energy hole problem effectively. Moreover, GRSUNDSOA also guarantees the full connectivity of network like the other three algorithms. Thus, GRSUNDSOA can improve the network lifecycle on the whole.

In practical UWSN environments, the free-floating sensors tend to change the network topology gradually due to factors such as waves, wind, and vorticity. As future work, we plan to study the effect of dynamic network topology changes on network coverage and connectivity. We also plan to further improve the deployment strategy of underwater wireless sensor network.

## Figures and Tables

**Table 1:** Simulation parameter settings.

Parameter Names	Value
*N*	[80, 160]
*R_s_*	40 (m)
*Rc*_max_	80 (m)
*α*	[1.4, 1.8]
*β*	0.25
*γ*	0.05
*th*	0.6
*v*	2.4 (m/min)
*p*	0.6 (W)
∆*r*	1 (m)
*a*	0.8
*b*	0.2

## Data Availability

Not applicable.

## Glossary

**Nomenclature**	**Definition**
GRSUNDSOA	Growth Ring Style based Uneven Node Depth-adjustment Self-deployment Optimization Algorithm
GR	Growth Ring
SRNs	Subtree Root Nodes
FSRNs	Forward Subtree Root Nodes
BNS	Basic Node Set
HD	Horizontal Distance
ED	Expect Distance
OD	Offset Distance
MBCUEB	Model Based on Coverage Utilization and Energy Balance
**Symbols**	**Definition**
*N*	The number of sensors deployed in network
*N*_connect_	The number of sensors that connect with sink node
*Rb*	The communication radius of the sensor nodes
*Rs*	The sensing radius of the sensor nodes
*Rc*_max_	The maximum communication radius of the sensor nodes
*Ns_i_*	The sensor node with ID *i*
*Ns* *_i_* *.Z*	The depth of *Ns_i_* under water
*Ns*	The sensor nodes set deployed in network
*M*	The entire monitoring area of UWSN
*Length*,*Width,Height*	*Length*—The length of the monitoring area; *Width*—The width of the monitoring area; *Height*—The height of the monitoring area
*U*(*x,y,z*)	A pixel in monitoring area
*P*_cov_(*x,y,z,Ns_i_* )	The probability that *U*(*x,y,z*) is covered by node *Ns_i_*
*dist*(*Ns_i_,Ns_j_*)	The Euclidean distance between *Ns_i_* and *Ns_j_*
*d*(*Ns_i_*,*Ns_j_*)	The HD between *Ns_i_* and *Ns_j_*
*Edist*(*Ns_i_*,*Ns_j_*)	The ED between *Ns_i_* and *Ns_j_*
*Div_Ns_*(*Ns_i_*,*Ns_j_*)	The OD between *Ns_i_* and *Ns_j_*
*dist*_max_	The maximum Euclidean distance of all nodes from sink node
*P*_cov_(*U*, *Ns*)	The probability that *U* is covered by *Ns*
*Q_i_*	The monitoring area of sensor node *Ns_i_*
*η*	The coverage rate of UWSN
*Con*	The connectivity rate of UWSN
*V*(*Ns_i_*, *Ns_j_*)	The volume of the overlapping area covered by *Ns_i_* and *Ns_j_*
$\tilde{P}(Ns_{i})$	The coverage utilization of *Ns_i_*
*g*	The number of GR
*Root_g_*	The *g*-th GR
*basenodes*	The current BNS
∆*r*	The step length for the set {*Rc*_max_, *Rc*_max_-∆*r*, …., *d*(*Ns_i_*,*Ns_j_*)}
*N*_sin_	The numbers of nodes within *Rc*_max_ from the sink node
*N*_avg._	The average number of neighbor nodes for all sensors
*W*	The energy consumption for network deployment
*D*	The total distance that all nodes move during the deployment process
*v*	The moving speed of nodes
*p*	The power of nodes
*S_g_*	The nodes within the *g*-th GR
*Ns_i_*.<*id*, *tree*, *leaf*, *fathe*, *root*, *processed*>	The attributions of *Ns_i_*. *Ns_i_*.*id*: ID of *Ns_i_*; *Ns_i_*.*tree* indicates whether *Ns_i_* joins the tree structure (1—Yes, 0—No); *Ns_i_*.*father*: the *id* of its parent node; *Ns_i_*.*root*: the root node of the tree to which *Ns_i_* is attached; *Ns_i_. processed* indicates whether *Ns_i_* has been calculated the diving depth (1—Yes, 0—No); *Ns_i_*.*leaf* indicates whether *Ns_i_* is a leaf node (1—Yes, 0—No).
*distnodes*	The ordered node set of *S_g_* according to *dist*(*Ns_i_*, Sink) from largest to smallest
*α*	The adjustment parameter for communication radius
*β*	The adjustment parameter for communication radius
*γ*	The adjustment parameter for constructing *Root_g_* from *S_g_*
*r*	The random number
*th*	A threshold for constructing *Root_g_* from *S_g_*
*μ*	A non-negative integer threshold
*δ*	The set maximum of GR
*Z_Ns_*_(*j*)_	The calculated depth of the basic node *Ns_j_*
*Candidate*(*Ns_i_*)	The dive candidate position points set of *Ns**_i_*
$N{s^{\prime }}_{ik}$	One of candidate position points in *Candidate*(*Ns_i_*)
*a*, *b*	The weights for MBCUEB, *a*+*b* = 1
*f*(*Ns_i_*)	The comprehensive optimal model of *Ns_i_* on coverage utilization and energy balance
